# Participant retention in a fully remote trial of digital psychotherapy: Comparison of incentive types

**DOI:** 10.3389/fdgth.2022.963741

**Published:** 2022-09-06

**Authors:** Isabell R. Griffith Fillipo, Michael D. Pullmann, Thomas D. Hull, James Zech, Jerilyn Wu, Boris Litvin, Shiyu Chen, Patricia A. Arean

**Affiliations:** ^1^ Department of Psychiatry and Behavioral Sciences, CREATIV Lab, University of Washington, Seattle, WA, United States; ^2^ University of Washington SMART Center, Seattle, WA, United States; ^3^ Research and Development, Talkspace, New York, NY, United States

**Keywords:** incentives, retention, digital health, randomized trials, depression

## Abstract

Numerous studies have found that long term retention is very low in remote clinical studies (>4 weeks) and to date there is limited information on the best methods to ensure retention. The ability to retain participants in the completion of key assessments periods is critical to all clinical research, and to date little is known as to what methods are best to encourage participant retention. To study incentive-based retention methods we randomized 215 US adults (18+ years) who agreed to participate in a sequential, multiple assignment randomized trial to either high monetary incentive (HMI, $125 USD) and combined low monetary incentive ($75 USD) plus alternative incentive (LMAI). Participants were asked to complete daily and weekly surveys for a total of 12 weeks, which included a tailoring assessment around week 5 to determine who should be stepped up and rerandomized to one of two augmentation conditions. Key assessment points were weeks 5 and 12. There was no difference in participant retention at week 5 (tailoring event), with approximately 75% of the sample completing the week-5 survey. By week 10, the HMI condition retained approximately 70% of the sample, compared to 60% of the LMAI group. By week 12, all differences were attenuated. Differences in completed measures were not significant between groups. At the end of the study, participants were asked the impressions of the incentive condition they were assigned and asked for suggestions for improving engagement. There were no significant differences between conditions on ratings of the fairness of compensation, study satisfaction, or study burden, but study burden, intrinsic motivation and incentive fairness did influence participation. Men were also more likely to drop out of the study than women. Qualitative analysis from both groups found the following engagement suggestions: desire for feedback on survey responses and an interest in automated sharing of individual survey responses with study therapists to assist in treatment. Participants in the LMAI arm indicated that the alternative incentives were engaging and motivating. In sum, while we were able to increase engagement above what is typical for such study, more research is needed to truly improve long term retention in remote trials.

## Introduction

The purpose of this study to is to compare two types of incentive models to retain an optimal sample size in a large scale, remote and sequential multiple assignment randomized trial (SMART) of digital psychotherapy. Because of challenges in the access to mental health services, the number of mental health interventions based in digital platforms has grown (1, 2). According to recent Banbury and National Institute of Mental Health Advisory Council Reports (3, 4), while there is evidence to support these use of digital treatment for mental health conditions, there is still a long way to go to understand which types of digital mental health tools are most effective. To investigate the efficacy of these tools, clinical trials will need to be done remotely to emulate how care would be delivered and accepted in its natural and entirely virtual context. Remote clinical trials offer a number of opportunities to accelerate the digital mental health field, including the ability to recruit very large samples (hundreds of people over days or weeks), to recruit historically under-represented populations, and to inexpensively collect objective data through passive sensing (geolocation) and active means (online surveying; (5–7). Furthermore, remote research addresses the common problem of recruitment into clinical research; analysis of global data from the Clinical Trials Database reveal that of those trials that were terminated, 55% were terminated due to low recruitment (8).

### Challenges with retention in randomized clinical trials

Although the benefits of remote trials are many, this novel approach to research introduces new methodological challenges that have yet to be considered. One of the biggest challenges to remote clinical research is participant retention. Failures to retain optimal numbers of participants is a threat to validity due to sample bias. Poor retention in remote research tends to be early and at high proportions, with the most successful retention rates (56%) being below what is commonly seen as optimal (5–7). High or “good” retention is considered to be 80% or better of the sample completing the entire study (9), with some methodologists allowing for 70%–75% retention to be adequate owing to the use of multiple imputation, Bayesian, and weighted approaches to give unbiased estimation (10). However, even with the use of statistical approaches to addressing missing data and sensitivity analyses to evaluate the robustness of the results, the validity of findings is still questioned (11).

In all randomized clinical trials, be they remote or face-to-face, participants are usually randomized to a treatment condition, rather than choosing the condition they may prefer. Research has found that those randomized to a condition they least prefer are more likely to drop out of the study early (12, 13). In face-to-face trials, this can be mitigated by a research team member who can address specific concerns participants may have in being randomized (5, 6). In remote trials, particularly very large trials, contact with a research team member is usually focused on technical issues and tends to be conducted over asynchronous secure messaging. Data suggests that lack of synchronous human connection results in lower motivation to continue research participation (5, 6). Even in internet-based treatment trials where participants are in communication with coaches or clinicians, study dropout rates are high, particularly in the early weeks of a trial (1). The challenges to retention may be even more acute with more complex randomized trial designs (3) such as sequential multiple assignment randomized trials (SMART), (14, 15). SMARTs have more than one potential randomization time-period in the design. This additional randomization adds 2 or more periods where the risk of drop out is high. Although one meta-analysis found that retention is potentially better for in-person SMART designs, it is unclear if this is the case for remote SMARTs (16, 17).

### Current data and recommendations for retention in remote clinical studies

There is a substantial literature on the use of various incentive types to engage people in remote survey research, adherence to digital interventions, but very little research into retention in remote clinical trials. A variety of incentive models exists and are largely based on reinforcement theory (18). These models include (but are not limited to) gamification, behavioral economics, and monetary incentive. Gamification methods include strategies that are meant to “hook” participants into participation. Gamification strategies often include the delivery of motivating GIFs or easter eggs (intentional inside jokes or messages), the use of leaderboards to instill competition, and earning points that can be used to unlock information or in-app benefits. Behavioral economic strategies include using social norms to motivate engagement (similar to the leaderboard concept), allowing various choices for incentives, return of information and status tracking, and establishing a lottery system for tangible incentives (money, devices) (19). These approaches have been variably successful in survey based and clinical trial research across age groups and populations. Of these methods studied, financial or monetary incentives are seen by participants as both ethical and necessary for long term retention (20, 21). In the limited data on retention methods for remote and randomized clinical trials, one meta-analysis of digital health studies with large remote samples found that providing a monetary incentive resulted in better overall retention than providing no monetary incentive, where no monetary incentive resulted in retention rates as low as 10% (7). This meta-analysis confirms previous research into participant preferences and suggestions for encouraging long term participation (22, 23). A recent study of monetary incentives for the Verily Mood Baseline Study, a 12-week remote passive sensing and daily survey study, found that large monetary incentives resulted in 83% retention over the course of 12 weeks (16).

The size of monetary incentives needs to be carefully considered both from an ethical, practical and data quality perspective. Although most bioethicists find some monetary incentive to be ethical, there is a point at which the amount offered could be seen as coercive, and the potential of coercion may vary by population economics; for instance $25 may seem a nominal amount for someone who is employed, but a sizable amount for someone who is not employed (25). The incentive sizes that were used in the above referenced Verily Baseline Mood Study were quite large for relatively little effort, the lowest incentive condition was $250.00 USD for 12 weeks of passive sensing and daily EMAs of less than 4 items. This incentive amount is more than most funders are willing to support. Finally, we have found in our own research that incentives as large as $90 USD for a 12 weeks participation attracts malicious actors, people who volunteer for a study for financial benefit, and who are not true representatives of the population of interest (17, 26). Thus, while there is preliminary support for the use of monetary incentives to retain remote samples into clinical research, the incentives that do appear to be effective are very high and may result in problems with ethics, economic practicality, and malicious acting.

The data from the few studies of alternative incentive models (gamification and behavioral economics) have been met with mixed acceptance by participants, although one meta-analysis found that gamification methods can increase intrinsic motivation to participate (27). To our knowledge, no one has looked at alternative to monetary incentives for remote and randomized clinical trials, although a meta-analysis of in-person trials found no significant impact on study retention for gamification or behavioral economics (28). Thus, while such methods may have the potential to retain samples in remote trials, more research is needed to determine the effectiveness of these methods on remote study retention.

Another option for study retention is to combine the use of low monetary incentive with alternative incentive strategies. Given the preference to receive monetary incentives for participation, methods including monetary incentive should be considered for remote clinical trials; to avoid the potential for coercion and malicious acting, these need to be perceived as low incentives. Thus, it is important to determine whether a combination of alternative, intrinsic incentives that participants believe are engaging (humor, motivational messaging, and return of information), with lower, traditional levels of monetary incentives can be as effective in retaining participants in a longitudinal study as high monetary incentives.

The implicit model we test in this paper examines the impact of these two types of incentives models. Study burden is a barrier to study completion; it represents the degree of effort required to overcome the inertia of not completing study measures. A high monetary incentive acts as an extrinsic motivator to overcome the impact of study burden. The more burdensome a study is, the higher the incentive needed. Therefore, the sense of adequacy of the monetary incentive should mediate the impact of incentive on study participation. Intrinsic motivation may also help reduce the sense of study burden, and this intrinsic motivation can be activated by alternative sources as described above (e.g., humor, motivational messaging, and return of information). The balance of study burden, extrinsic motivation *via* incentives, and activation of intrinsic motivation then leads to study engagement and retention.

The purpose of this paper is to report on the findings from a feasibility study of a SMART to determine the impact of incentive type (high monetary incentives or combined low monetary incentives and alternative incentives) on study retention. Here we defined retention as weekly completion of primary outcome measures, the completion of the tailoring assessment to determine need for re-randomization and subsequent retention post re-randomization. Based on our foundational research into the preferences of research participants on these platforms, we hypothesize that the use of the combined incentives, such as information and GIFs, coupled with low monetary incentives would lead to retention rates comparable to high monetary incentive alone (1, 7, 22–24, 29). We also explored whether participant sense of burden of the research study, adequacy of payment, and intrinsic rewards were associated with increased study participation.

## Materials and methods

This study used data collected during the pilot phase of a fully remote SMART design comparing the effectiveness of message-based psychotherapy, tele-psychotherapy, and the combination of these delivery platforms. One aim of the pilot phase was to determine the optimal incentive strategy to use during the trial. This study was reviewed by the University of Washington Institutional Review Board and approved on May 29, 2020. The study period was between February 26 and July 25 of 2021.

### Participants

Two-hundred and fifteen participants, 18 years old and older, living in the U.S, were recruited from a screening platform hosted by Mental Health America, a US based mental health advocacy program. To be eligible, participants had to have either a score of 10 or greater on the PHQ-9 at screening or have received a diagnosis of depression from a Talkspace intake clinician. Participants in this study were representative of the typical patient seeking care through an online platform for symptoms of depression, as is evident from research from other studies testing similar platforms (30–33). Exclusion criteria were a history of bipolar disorder, psychosis, and active suicidal ideation; participants who had active psychosis or suicidal ideation were referred to intensive care. Table 1 displays sample characteristics.

**Table 1:** Demographics and Descriptives by Condition.

		Condition	
	Overall *N* (%)	Low incentive *n* (%)	High incentive *n* (%)
Sample	215 (100)	106 (49.3)	109 (50.7)
Gender			
Female	171 (79.5)	80 (75.5)	91 (83.5)
Male	30 (14.0)	19 (17.9)	11 (10.1)
Transgender Male	1 (0.5)	1 (0.9)	0 (0.0)
Something else	11 (5.1)	6 (5.7)	5 (4.6)
Prefer not to say	2 (0.9)	0 (0.0)	2 (1.8)
Latinx Ethnicity			
Latinx & American Indian & Asian & Black & Native Hawaiian	1 (0.5)	1 (0.9)	0 (0.0)
Latinx & Other multiracial	4 (1.9)	3 (2.8)	1 (0.9)
Latinx and Asian	1 (0.5)	1 (0.9)	0 (0.0)
Latinx and Black	4 (1.9)	3 (2.8)	1 (0.9)
Latinx and White	34 (15.8)	18 (17.0)	16 (14.7)
Latinx only	26 (12.1)	10 (9.4)	16 (14.7)
Not Latinx	145 (67.4)	70 (66.0)	75 (68.8)
Race			
Asian	17 (7.9)	10 (9.4)	7 (6.4)
Black or African American	20 (9.3)	14 (13.2)	6 (5.5)
White or Caucasian	128 (59.5)	61 (57.5)	67 (61.5)
American Indian, Asian, Black, & Native Hawaiian	1 (0.5)	1 (0.9)	0 (0.0)
American Indian, Black, & White	1 (0.5)	0 (0.0)	1 (0.9)
Asian & Black	1 (0.5)	0 (0.0)	1 (0.9)
Black & Caucasian	2 (0.9)	1 (0.9)	1 (0.9)
Other multiracial, unspecified	23 (10.7)	11 (10.4)	12 (11.0)
Prefer to self-describe	15 (7.0)	7 (6.6)	8 (7.3)
Prefer not to say	7 (3.3)	1 (0.9)	6 (5.5)
Marital status			
Married or partnered	68 (31.6)	33 (31.1)	35 (32.1)
Divorced	9 (4.2)	5 (4.7)	4 (3.7)
Separated	4 (1.9)	3 (2.8)	1 (0.9)
Widowed	1 (0.5)	1 (0.9)	0 (0.0)
Single, never married	132 (61.4)	63 (59.4)	69 (63.3)
Prefer not to say	1 (0.5)	1 (0.9)	0 (0.0)
Education			
Some high school/less than high school diploma	2 (0.9)	1 (0.9)	1 (0.9)
High school diploma/GED	22 (10.2)	12 (11.3)	10 (9.2)
Some college	73 (34.0)	36 (34.0)	37 (33.9)
Associate’s degree	19 (8.8)	10 (9.4)	9 (8.3)
Bachelor’s degree	64 (29.8)	29 (27.4)	35 (32.1)
Master’s degree	27 (12.6)	14 (13.2)	13 (11.9)
Professional degree	5 (2.3)	2 (1.9)	3 (2.8)
Doctoral Degree	3 (1.4)	2 (1.9)	1 (0.9)
First time in therapy	114 (52.8)	56 (52.3)	58 (53.2)
	**M (SD)**	**M (SD)**	**M (SD)**
PHQ baseline total	17.7 (4.4)	17.3 (4.3)	18.2 (4.4)
Age	29.7 (9.5)	29.2 (8.8)	30.2 (10.2)

There were no significant differences between conditions on any demographic and descriptive variables.

**Figure 1:**
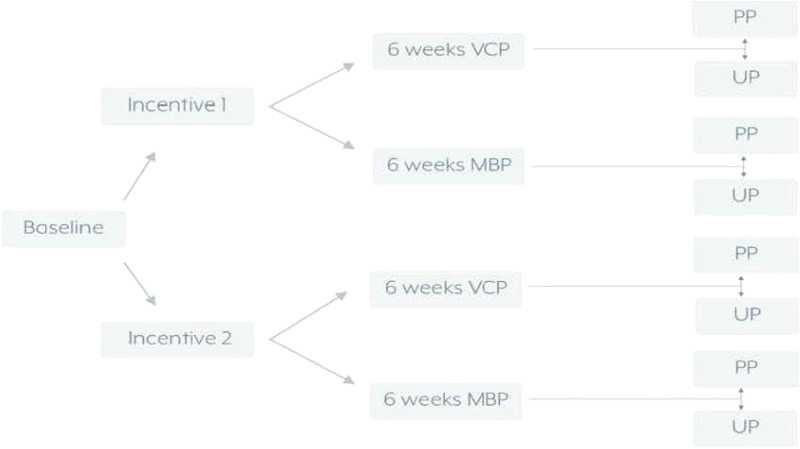
Randomization scheme for the study.

**Figure 2:**
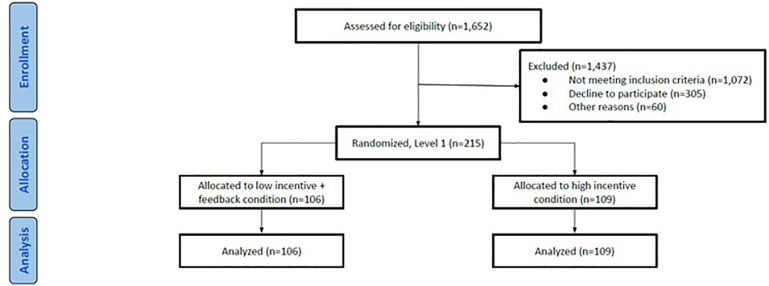
CONSORT Table for incentive condition assignment.

Figure 1 presents the randomization scheme for the pilot study; Figure 2 presents the CONSORT Table showing randomization to incentive condition. Participants were first randomized to incentive condition and then to a treatment condition. Of the overall sample of 215, there were 106 (49.3%) participants randomly assigned to the low-monetary plus alternative incentive condition and 109 (50.7%) assigned to high monetary incentives. There were no statistically significant differences between incentive or treatment conditions on any demographic or descriptive variables. The sample was largely female (171/215, 79.5%). Participants were White (128/215, 59.5%), Latinx only (26/215, 12.1%), Black (20/215, 9.3%) or Asian (17/215, 7.9%). Most participants were single, never married (132/215, 61.4%) or married (68/215, 31.6%). Most had attended some college but had not yet earned a degree (73/215, 34%) or had a college degree (64/215, 29.8%). About half had never received therapy before (114/215, 52.8%). The average PHQ-9 total score at baseline was 17.7, in the moderately severe range. The average age was 29.7.

### SMART design

This study was meant to test the feasibility of conducting a remote SMART design of on-demand message-based care alone compared to weekly psychotherapy sessions conducted over secure video conferencing, through Talkspace, a digital mental health company that provides psychotherapy to people throughout the 50 United States. The SMART included an initial randomization of participants to 12 weeks of intervention delivered either *via* secure messaging or video chat. At week 5 of treatment, we used the Patient Health Questionnaire-9 (PHQ9) as our tailoring variable to determine if participants were responding adequately to their assigned condition. If participants were responding well, they were not randomized further; if they were not responding, participants were randomized to receive one of two augmentation strategies, weekly video conferencing with message-based care or monthly video conferencing with message-based care [Details of the intervention conditions for this study are described in a protocol paper (34)]. Thus, the PHQ9 completion before week 5 and at week 12 (end of treatment) were two important time-periods where retention/assessment completion was important.

### Incentive conditions

Prior to being randomized to treatment conditions, participants were randomized to one of two incentive conditions, (1) high monetary incentive (HMI; $125USD), and (2) combined low monetary and alternative incentive (LMAI; $75USD). The two monetary incentive values were based on a meta-analysis of various incentives (7) and on user-centered design research asking a representative sample of 20 US dwelling adults with depression which type of incentive was viewed as fair (35). Although participants did interact with study therapists as part of the treatment protocol, interaction with the study team was limited to informed consent, technical assistance, reminders, and thanks for participation.

The LMAI engagement condition included additional, in app messages of encouragement for completing daily assessments, facts about depression, humorous GIFs after completing surveys, and prompts to reflect on their responses to their daily activity surveys and how that compared to their mood. The participant engagement method used in the LMAI condition was co-designed with representative participants at the University of Washington ALACRITY Center (UWAC), using Human Centered Design and User Experience Research methods, that employed A/B testing and interactive design with 20 participants suffering from depression to ensure strategies were useful, meaningful, understandable, and engaging, and to determine what incentive amount was deemed to be the lowest yet the most fair compensation amount (23).

For both groups, payment was directly tied to their completion of weekly surveys combined with their completion of the baseline and exit surveys, and ultimately calculated and distributed to each participant after each 4 weeks of participation, resulting in 3 total payments. In the HMI condition, participants earned $21 for completing the baseline survey, and the LMAI condition participants earned $12. An extra incentive of $8 was designated for the HMI condition participants after completing the exit survey. LMAI condition participants earned a bonus of $3. Participants in the HMI condition earned $8 for completing each weekly survey, and participants in the LMAI condition earned $5 for completing each weekly survey. Participants were paid every 4 weeks, resulting in a total of 3 payments which were distributed in the form of Amazon gift codes by email. See supplemental materials for details on the user centered design strategies and findings, as well as example of feedback and engagement strategies (35).

### Assessments

Details on assessment for this study are provided in a protocol paper (34). For the purposes of this paper, we describe when measures were deployed, anticipated length of on-line assessment and a description of the exit survey. The aims of the current study do not include analyses of the clinical outcome measures.

#### Screening

Interested individuals created an account with Talkspace and completed a 5-minute screening survey on Talkspace's website to provide basic demographic information and complete the PHQ-9. A research coordinator reviewed the screening survey for eligibility, and *via* email, notified participants of their eligibility and sent a consent form.

#### Baseline assessment

After providing consent, participants completed a demographic questionnaire and the following measures *via* a REDCap survey on their mobile device or computer: (1) the Major Depressive Episode Screener (36, 37) which is an on-line assessment of major depression, (2) the 9-item Patient Health Questionnaire (PHQ-9) (38), (3) The Social Life and Family Life Scales of the Sheehan Disability Scale (SDS) (39), (4) 7-item Generalized Anxiety Scale (GAD-7) (38), (5) the NIAAA Alcohol Screening Test (40) and (6) the IMPACT assessment of mania and psychosis (41). The baseline assessment package required approximately 15 min to complete.

#### Weekly assessments

Every week, participants were administered the PHQ-9, the Sheehan Disability Assessment Scale, and GAD7. We also administered the Patient Global Improvement Scale, a five-item measure of participant perception of improvement since beginning treatment. Participants are asked to rate their improvement since using the apps. Specifically, participants are asked, “since starting treatment, I feel that I am: (1) much worse (2) worse (3) no different (4) improved (5) much improved. The weekly assessment package required 10 min to complete.

#### Exit survey (study satisfaction, burden, intrinsic motivation, adequacy of incentive, feedback on engagement strategies)

The final administration, Week 12 survey, included the usual weekly items along with an exit survey. Overall study satisfaction was collected *via* a single item “How satisfied were you in this study overall” with response options on a five-point scale, from 1 = Very unsatisfied to 5 = Very satisfied. Burden of completing weekly measures was collected *via* a single item, “How burdensome did you find completing the daily surveys” with response options on a five-point scale from 1 = Not burdensome to 5 = Very burdensome. Intrinsic motivation to participate in the weekly surveys was collected *via* a single item, “Some people find getting weekly surveys to be an interesting experience, an opportunity to learn more about yourself. Is this true for you or not?” with dichotomous response options, 0 = False, 1 = True. Adequacy of incentive was collected *via* a single item, “It is typical in the US to pay research participants for completing surveys. Did you feel the amount you were compensated for participation to be…” with response options on a five-point scale, 1 = Too low, 2 = Low but fair, 3 = The right amount, 4 = Too much but fair, 5 = Too much.

Participants in the LMAI condition were also asked to indicate which engagement strategies they liked or disliked. Participants were able to respond using fixed choice options and with open-ended answers regarding their experiences and suggestions for improving each feature. The open-ended questions were:

• “Please give us more information about your choices and any ideas for improving this feature (facts),”
• “Please give us more information about your choices and any ideas for improving this feature (insights),”
• “Please give us more information about your choices and any ideas for improving this feature (GIFs),”
• “Finally, if there was a way for us to make the experience more engaging, what do you recommend?,”
• “Were there other kinds of insights you would have liked to have seen,”
• “Did you get a chance to think about ideas for improving the experience you just went through? Jot them down here,” and,
• “What ideas do you have to make participation in a study such as this one more engaging?”

#### Reminders to complete surveys

Participants were reminded to complete weekly surveys once *via* short message service (SMS) after 24 h if the participant did not complete the weekly survey in the first day after their first SMS notification.

#### Technical assistance communications

Participants were encouraged to send an email with any questions regarding the study or if they encountered issues with their therapist, the Talkspace platform, or surveys. The study team would respond to concerns within one business day.

#### Retention outcomes

For data analysis, participant retention outcomes were assessed continuously as total weekly assessments completed and as percentage of participants completing the tailoring variable of the PHQ9 at 3, 4, and 5 weeks, and end of treatment assessment PHQ9 at 10 and 12 weeks

### Analyses

Analyses were designed to answer the primary research question focusing on retention in four different ways. First, for descriptive purposes, we computed the proportion of weekly assessments completed, defined as completion of the PHQ-9, for each of the 12 weeks of the study, overall and by condition. Odds ratios were computed for the LMAI condition as compared to the HMI condition. An *a priori* power analysis for a sample size of 100 participants in each condition was set to detect an odds ratio of 2.1, equivalent to a Cohen's *d* of .41 or a 12-percentage point difference in proportion of participants completing the PHQ. This assumes an 85% response rate from the best-responding condition.

Second, we tested whether conditions differed on their overall rates of completion of the PHQ-9. A mixed effects regression specifying a Poisson distribution, including random effects to adjust standard errors to account for nesting of client within therapist, was computed to compare groups on the proportion of total weekly PHQ-9 measures completed. We used Restricted Maximum Likelihood estimation with an unstructured covariance matrix.

Third, we analyzed whether there were differences by condition in the proportion of participants who provided at PHQ for weeks 3, 4, or 5, which are relevant for tailoring for the SMART trial, using a crosstabulations with chi-square test.

Fourth, we analyzed the length of time until study dropout, with dropout defined as the last PHQ-9 completed by a participant. A Cox regression time-to-event analysis was computed to identify the number of weeks until study dropout. As length of time until study dropout was of primary interest, we explored this further using other descriptive information. Gender, age, and PHQ baseline score were included as covariates in separate Cox regression models with condition x covariate interaction terms to test for moderation. Cox regression curves were tested using the log-rank test.

Other analyses focused on elements of the study that may have also been impacted by condition. Independent sample t-tests were computed to test for differences in participant-reported study satisfaction, study burden, and sense of adequacy of incentive amount. A crosstabulation with chi-square test was computed to test whether condition was associated with post-study reports of intrinsic motivation for study participation. Frequencies, means, and standard deviations were computed on ratings of insights, facts, and gifs. We computed correlations and point-biserial correlations to test whether study satisfaction, study burden, adequacy of incentive amount, and intrinsic motivation were associated with the total number of PHQs completed, and whether burden was associated with sense of adequacy of incentive.

Qualitative analyses were conducted on open-ended items related to the alternative incentive engagement strategies in the LMAI condition. A coding team of three researchers read each responders' answers to the open-ended questions and met to develop an initial codebook of themes and topics found in these responses, with each code universal to all questions. Each researcher then independently re-read and coded all comments, applying up to five codes for each response. To examine interrater reliability among the three coders, Cohen's *κ* values were calculated for each rater pair, followed by individual item mean and an overall mean (42). The mean overall Cohen's *κ* was 0.66, within the realm of substantial agreement (43). To improve upon this, final codes were applied *via* team consensus for each code with disagreement. In three instances where the raters could not reach full consensus on codes, majority rule determined the final code.

## Results

Weekly proportions of participants completing PHQ-9s are displayed in Table 2. Raw proportions indicate that the LMAI condition had higher PHQ-9 response rates for the first five weeks and the HMI had higher response rates for weeks 7 to 11. Odds ratio statistics and 95% confidence intervals reveal that the baseline week had a significantly higher proportion of LMAI users completing the PHQ-9. However, these cell sizes are small, which can inflate odds ratio statistics. Week 5 was also slightly, and significantly, in favor of the LMAI group. The odds ratio for the average of all weeks was 1.29 (95% CI = 0.94, 2.24), equivalent to a Cohen's *d* of 0.14. This odds ratio was lower than the *a priori* power analysis expectation of 2.1 (Cohen's *d* of .41).

**Table 2:** Weekly measure completion rates and odds ratios.

	Overall *N* (%)	Low incentive *n* (%)	High incentive *n* (%)	Odds ratio	OR 95% CI
*n*	215 (100)	106 (100)	109 (100)	–	–
Baseline	196 (91.2)	100 (94.3)	96 (88.1)	6.77	5.26, 8.29
Week 1	165 (76.7)	80 (75.5)	85 (78.0)	1.03	0.37, 1.68
Week 2	161 (74.9)	81 (76.4)	80 (73.4)	1.40	0.76, 2.04
Week 3	151 (70.2)	78 (73.6)	73 (67.0)	1.60	1.00, 2.21
Week 4	145 (67.4)	75 (70.8)	70 (64.2)	1.55	0.96, 2.14
Week 5	142 (66.0)	74 (69.8)	68 (62.4)	1.59	1.01, 2.18
Week 6	141 (65.6)	69 (65.1)	72 (66.1)	1.07	0.50, 1.65
Week 7	129 (60.0)	62 (58.5)	67 (61.5)	0.97	0.42, 1.53
Week 8	133 (61.9)	64 (60.4)	69 (63.3)	0.98	0.42, 1.54
Week 9	125 (58.1)	59 (55.7)	66 (60.6)	0.89	0.34, 1.44
Week 10	120 (55.8)	54 (50.9)	66 (60.6)	0.73	0.19, 1.28
Week 11	114 (53.0)	55 (51.9)	59 (54.1)	0.99	0.45, 1.53
Week 12	121 (56.3)	60 (56.6)	61 (56.0)	1.12	0.58, 1.67
Mean	–	–	–	**1** **.** **29**	**0.94, 2.24**

A mixed effects regression models found no differences between conditions on the average proportion completing the PHQ-9 (intercept = 0.591, intercept SE = 0.032, intercept *p *< .001; high incentive estimate = .003, SE = .045, *p *= .931). An average of 65% of those in the LMAI condition and 66% of those in the HMI condition completed a weekly measure over each of the 12 weeks. To assess who would need to be randomized to one of the combined conditions, participants were required to complete a PHQ-9 in weeks 3, 4, or 5 of the study. Approximately 75% of the sample completed a PHQ-9 during these weeks, with no difference in proportion completed between the two incentive arms (Low incentive: 81/106, 76.6%; High incentive: 82/109, 75.2%, X^2^_(1)_ = .006, *p *= .936).

**Figure 3:**
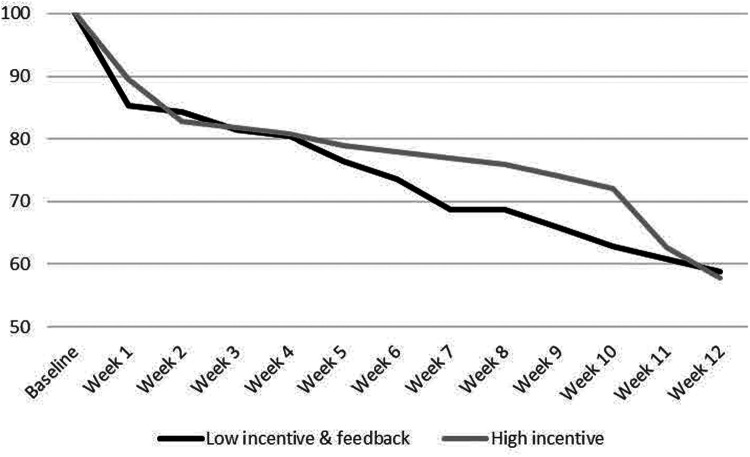
Length of Time until Study Drop.

Figure 3 displays a time-to-event curve of the length of time until dropout. This figure indicates possible retention differences in favor of the HMI group, with a 9-point difference in Week 10. However, a Cox regression time-to-even analysis with random effects for nesting by therapist found no significant difference between conditions in the overall length of time until dropout (Est = − 0.05, OR = .95, *p *= .80). Cox regression analyses found that gender significantly predicted retention, with males ending participation more quickly (Est = 0.42, OR = 1.53, *p* = .042). Not significantly associated with retention were baseline PHQ-9 score (Est = .042, OR = 1.04, *p *= .203) or age (Est = −0.02, OR = 0.98, *p *= .125). There were no significant condition x moderator effects for gender (Est = −0.48, OR = 0.62, *p *= .379), age (Est = −0.01, OR = 0.98, *p* = .583) or baseline PHQ-9 score (Est = 0.04, OR = 1.04, *p* = .499).

### Exit surveys

Note that the exit survey was only completed by participants who completed the Week 12 survey. A total of 114 participants responded to the exit survey, 56 (52.8%) in the LMAI condition and 59 (54.1%) in the HMI condition. There were no significant differences between conditions on mean reported study satisfaction, study burden, intrinsic motivation to participate, or adequacy of incentive (see Table 3). However, Table 4 displays significant correlations between the number of PHQs completed and reported burden of completing weekly surveys (*r* = −.329, *p* < .001), intrinsic motivation to participate (point-biserial *r* = .216, *p *= .021), and ratings of adequacy of incentive (*r* = .268, *p *= .004). There was not a significant correlation between number of PHQs completed and overall study satisfaction (*r* = .098, *p *= .310). Perceived survey burden was associated with adequacy of incentive (*r =* −.341, *p *< .001).

**Table 3:** Mean reported study burden and satisfaction by incentive conditions.

	Condition	Mean	SD	Cohen’s *d*	*p*
Overall study satisfaction	Low	3.82	0.98	.078	0.680
	High	3.90	1.04		
Burden of weekly surveys	Low	1.80	0.95	−.003	0.985
	High	1.80	1.01		
Adequacy of incentive compensation	Low	2.30	0.71	−.075	.689
	High	2.36	0.69		
	** **	**N**	**%**	***χ* ^2^**	***p***
Participating is intrinsically rewarding	Low	44	78.6	.831	.362
	High	42	71.2		

**Table 4:** Correlation table of condition, mechanisms, and number of PHQs completed.

	Satisfaction	Burden	Intrinsic motivation	Adequacy of incentive	Number of PHQs
Condition	−054	.004	−.085	.038	−.113
Satisfaction		−.118	.164	.205*	.098
Burden			−.285**	−.341**	−.329**
Intrinsic motivation				.190*	.216*
Adequacy of incentive					.268**

* *p* < .05.

** *p* < .01.

Participants in the LMAI condition completed additional survey questions about the non-monetary engagement strategies (see Table 5). A majority of participants rated the facts, insights, and gifs positively, with 94.5% rating the gifs as fun and 50% rating the facts and insights as useful.

**Table 5:** Ratings of insights, facts, and gifs provided by low-incentive group.

	Facts		Insights		Gifs	
	*N*	%	*N*	%	*N*	%
Useful	28	50.0	28	50.0	N/A	N/A
Not useful	2	3.6	4	7.1	N/A	N/A
Interesting	29	51.8	24	42.9	N/A	N/A
Annoying	0	0.0	0	0.0	0	0.0
Engaging	18	32.1	17	30.4	12	21.4
Something to look forward to	13	23.2	10	17.9	13	23.2
Unnecessary	1	1.8	0	0.0	6	10.7
Depressing	N/A	N/A	1	1.8	N/A	N/A
Activating	N/A	N/A	5	8.9	N/A	N/A
Fun	N/A	N/A	N/A	N/A	44	94.5
Childish	N/A	N/A	N/A	N/A	3	5.4

Qualitative coding generated three major themes from the LMAI condition regarding the alternative engagement strategies:

1) **Alternative incentive elements were rewarding.** Participants had overall positive feedback on all engagement elements. Representative quotes from open-ended questions were “the fact notifications were very beneficial [and] quite memorable” and “[the insight notifications] helped make the study feel like more of an experience than just being a lab rat”. GIFs in particular were seen as highly positive in that participants felt they were a member of the study team rather than a source of data, as this representative quote illustrates: “I love gifs, the more the better. Especially after having to access how you’re doing, which may not be pleasant. And it was like positive reinforcement”.
2) **Increased communication, data sharing and tracking would increase engagement.** Participants had suggestions for additional engagement strategies, including the wish for increased communication and data sharing between the research team, treatment therapist, and each participant. Participants wanted to have access to the data they were providing each day (“I would like to be able to see my results. Like how does my sleep correlate to my mood”), expressed interest in the tracking of and access to data metrics like mood, sleep, and activity levels (“Maybe a text box with the surveys so that we can detail out our experience that specific day in ways that isn’t offered by the questions about sleep and mood”), and wanted their therapists to also have access to this data so that they could adapt their provision of treatment accordingly, (“…if our therapists were able to see our answers […] maybe they could touch on things that might be affecting our mental health.”)
3) **Increased personalization would increase engagement.** Participants mentioned the need for greater personalization, such as more reflection exercises about new activities (“maybe a personal question about something new we did/tried that week? It would get people thinking about things they accomplished/motivate them to accomplish at least one thing each week”), or a mood tracker based on study survey responses (“I think a daily emotional check in that patients could use and keep for themselves could be helpful. Kind of like a bullet journal. Not a mandated thing, but something that [is] an option”).

## Discussion

This study provides important insights into the methodological challenges of conducting large scale, remote randomized clinical trials and SMARTs, in particular methods to encourage participant retention to optimal levels. We were able to confirm our hypothesis that low-monetary incentive coupled with alternative incentives would result in similar retention rates to high monetary incentives. There was some indication that low monetary incentives coupled with alternative engagement strategies was associated with better measure completion rates early in the study, and that high monetary incentive was associated with longer term study retention, but these differences generally attenuated over the course of the 12 weeks of the study. Over all 12 weeks, the odds ratio and effect size were small and consistent with meta-analyses from in-person clinical trials, which range from -.11 to .14 depending on the incentive approach (28).

While our hypothesis about similar retention rates was supported, our implicit hypothesis that the use either incentive type would result in optimal retention was not supported (44). The overall completion rates for both groups at week 12 were just under 60%, which is suboptimal but much better than rates of engagement in other remote mental health clinical trials (5–7). Most of the studies, which are largely simple randomized clinical trials, tend to have very high initial drop out, with retention into remote research is notoriously low; While upwards of 3,000 people agree to participate in a trial, many drop out of treatment early, resulting in user sample sizes of 200–300 patients, a 10% retention rate (5, 13, 35, 45, 46). While the low-incentive plus alternative incentive condition holds promise as a method for improving research engagement, the retention rates are not ideal for data quality and analysis.

Surprisingly, there were no differences between incentive conditions in their ratings of adequacy of incentive, study satisfaction, or sense of study burden. Because participants lack information such as the “average” study burden or incentive provided for participating in trials, participants may not be able to easily judge the fairness of monetary incentive amounts. Indeed, even the difference between $75.00 USD and $125.00 USD may not be seen as vastly different, and for some populations, $75.00 USD may be seen as a high incentive. Although our participants rated these incentives as fair, and our past research suggest that $75.00USD for 12 weeks of participant is the smallest amount people feel is fair, we were not able to determine if participants found the monetary incentives as equally far, and if they felt being paid $5.00 vs. $8.00 a week for completing surveys to be substantially different. We did find that higher ratings of fairness of incentive and lower survey burden were associated with greater number of PHQs completed and that increased survey burden was associated with lower ratings of the adequacy of incentive. It may be that these variables (sense of adequacy of incentive and research burden) are important mechanisms of the hypothesized link between incentive and measure completion, but the incentive conditions in this study were not effectively targeting those mechanisms. Intrinsic motivation was negatively associated with study burden and positively associated with number of PHQs completed yet, condition was not associated with intrinsic motivation. While this study attempted to applied methods to activate intrinsic motivation based on research we had conducted prior using Human Centered Design methods, it does not appear that intrinsic motivation was strongly activated by the strategies we used in the low-monetary with alternative incentives condition. Further research should investigate methods for enhancing intrinsic motivation to continue participation in research as well as determine optimal study burden to incentive match.

Data from our qualitative analysis into the value of incentives, intrinsic motivational strategies and suggestions from the participants may shed some light as to how to better improve retention in remote studies. While participants in the low-monetary plus alternative incentive arm rated the alternative engagement strategies favorably, they also expressed interest in being offered visual methods for tracking their survey responses throughout the study, and to have these data made available to clinicians in the study. A recent meta-analysis of RCTs of apps targeting depressive symptoms in adults found that dropout rates were lower in studies that offered human feedback and in-app mood-monitoring features (1). However, some caution is needed in clinical trials that focus on intervention effectiveness or in pragmatic trials, where the only information provided to clinicians are those that are normally available to them in their usual workflow. Providing clinicians with more information that is normally available to them has the potential to inflate the clinical value of an intervention (47).

As a final note, we highlight here that even with the ability to recruit large samples in a short time frame, we found that not only were men very under-represented in the sample, but they were also more likely to terminate study participation earlier. While in general, men are overrepresented in biomedical research, they are historically under-represented in mental health research (48), and are less likely to use psychosocial treatment (49). Methods to engage and retain men in mental health research needs to be better explored to ensure researchers are able to study gender differences in treatment response, a requirement of most scientific funding agencies (50). This finding suggests that there may be differential impacts of incentives on certain populations, a question that should be explored in future research.

### Limitations

The findings from this study should be viewed with the following limitations in mind. First, we did not have sufficient data to understand why 10% of participants who completed week 10 assessments failed to complete their week 12 assessment. As we found no differences in clinical outcomes in mood at 10 weeks, the reason for this drop out at the very end of treatment is unclear. Second, the exit interview was conducted with study completers and did not include people who dropped out of the project. Therefore, the generalizability of these participants’ recommendations may be limited to those who are naturally motivated to complete a project. Third, a prior remote clinical survey with daily and weekly assessments of mood had 80% retention at 12 weeks when paying participants $135 USD for participation (24). Our high monetary incentive arm was $10 less and the other condition, but the difference may have been sufficient to impact our 12-week retention outcomes. More research should look at varying levels of incentives to ascertain an incentive sweet spot for studies if this nature. Fourth, while we varied the dollar amount in the incentives, we did not vary the timing of incentives. It may be that more frequent payment during the study (every week rather than once a month) may have a more motivating impact. Fifth, while this project is a longitudinal study, most trials attempt to retain participants for 3 to 6 months after treatment to ascertain the long-term effects of clinical outcomes. We suspect, based on our data, that retention post intervention phase may worsen over time. We are not able to comment on the impact of these methods for improving retention during treatment follow-up phases. Fourth, our data on survey burden, intrinsic motivation, and adequacy of incentive was restricted to the 53% of participants who responded to the Week 12 survey; there may have been different, possibly stronger, findings were we able to survey those who had stopped responding to measures. Finally, the sample is limited to one recruited from the US. While it is a diverse sample, it is important to note that perspectives about research participation in the US may be quite different than participation in other countries. Two studies also found that engagement differed depending on different participant characteristics like gender, age, and other sociodemographic characteristics due to personal preference and behaviors (51), but also factors like the availability of certain digital tools in a population and the appropriateness of a given engagement tool for different participant groups (29). Different forms of engagement may be more helpful and impactful to some people than others, and research should continue to investigate the personalization of incentives further. We advocate conducting Human Centered Design work with representative samples of a future project to ensure the engagement strategies and research burdens are tailored to the population in need.

## Conclusion

We were able to demonstrate that combining low-monetary incentives with alternative incentives results in overall retention rates similar to retentions rates in people receiving high monetary incentive. However, by the end of the study, retention rates are still below what is considered statistically optimal. Compared to past research demonstrating very low long-term retention in remote clinical trials, some financial or other incentive is better than no incentive, as evidenced by the ability to retain more than is typical in such trials, but more work is needed to determine how to improve optimal retention. Our next steps are to determine if the combination of high incentive with alternative incentives can improve overall retention, weekly completion of assessments, and completion of tailoring assessments to an optimal level.

## Contribution to the field

Remote clinical trial methods for the study of digital mental health tools are still in its nascence. Remote trials are appealing in that they can (1) reach large numbers of people with conditions of interest in a relatively short period of time, (2) reduced burden on the study participant, owing to the ability of participants to complete research tasks at on their own time and schedule and (3) can increase sample diversity, which has been a challenge for traditional studies. When studying digital mental health, particularly existing platforms, trials should emulate as much as possible the context in which these interventions are delivered, which is remotely, with minimal human contact beyond what is needed in the intervention. However, remote trials currently struggle with long-term retention, and the best methods to improve retention are not well studied. This paper presents data on a randomized trial comparing two participant incentive models for a 12-week, sequential multiple assignment randomized trial of message-based psychotherapy vs. tele-psychotherapy for depression. The results of this study find generally equivalent retention by incentive type, better retention than is typical with such trials, but still below what is considered optimal for data quality.

## Data Availability

The raw data supporting the conclusions of this article will be made available by the authors, without undue reservation.
